# Treatment with solubilized Silk-Derived Protein (SDP) enhances rabbit corneal epithelial wound healing

**DOI:** 10.1371/journal.pone.0188154

**Published:** 2017-11-20

**Authors:** Waleed Abdel-Naby, Brigette Cole, Aihong Liu, Jingbo Liu, Pengxia Wan, Ryan Schreiner, David W. Infanger, Nicholas B. Paulson, Brian D. Lawrence, Mark I. Rosenblatt

**Affiliations:** 1 Department of Biomedical Engineering, Cornell University, Ithaca, New York, United States of America; 2 Department of Ophthalmology, Weill Cornell Medical College, New York, New York, United States of America; 3 Department of Research, Silk Technologies, Ltd., Plymouth, Minnesota, United States of America; 4 Department of Ophthalmology and Visual Sciences, University of Illinois at Chicago, Chicago, Illinois, United States of America; Cedars-Sinai Medical Center, UNITED STATES

## Abstract

There is a significant clinical need to improve current therapeutic approaches to treat ocular surface injuries and disease, which affect hundreds of millions of people annually worldwide. The work presented here demonstrates that the presence of Silk-Derived Protein (SDP) on the healing rabbit corneal surface, administered in an eye drop formulation, corresponds with an enhanced epithelial wound healing profile. Rabbit corneas were denuded of their epithelial surface, and then treated for 72-hours with either PBS or PBS containing 5 or 20 mg/mL SDP in solution four times per day. Post-injury treatment with SDP formulations was found to accelerate the acute healing phase of the injured rabbit corneal epithelium. In addition, the use of SDP corresponded with an enhanced tissue healing profile through the formation of a multi-layered epithelial surface with increased tight junction formation. Additional biological effects were also revealed that included increased epithelial proliferation, and increased focal adhesion formation with a corresponding reduction in the presence of MMP-9 enzyme. These *in vivo* findings demonstrate for the first time that the presence of SDP on the injured ocular surface may aid to improve various steps of rabbit corneal wound healing, and provides evidence that SDP may have applicability as an ingredient in therapeutic ophthalmic formulations.

## Introduction

Annually, over 50 million people worldwide suffer from blindness due to corneal disease, and nearly 2.5 million new eye injuries occur each year in the United States, representing the greatest ocular environmental health problem today [1]. In addition, it is estimated that annually, another 337 million people suffer from the debilitating symptoms of dry eye disease worldwide, representing another significant threat to the ocular surface [2]. The current standard of treatment of ocular injury and dry eye disease involves the use of therapeutic eye drop formulations, such as steroids to reduce inflammation, antibiotics to thwart infection, and artificial tears containing lubricants to enhance comfort [3]. While eye drops offer ease of administration and wide accessibility, current therapeutic approaches do not directly enhance tissue regeneration, may inhibit the wound healing process with chronic use, or offer transient relief from symptoms [4]. For severe wounds, the application of human placental amniotic membrane to the injured ocular surface has been used to facilitate regeneration [5]. Although this approach has proved effective, amnion has restricted use to severe pathologies due to the required extensive technical skill to apply, risks disease transmission due to donor origins, and varies in both material consistency and therapeutic efficacy [6]. Thus, clinical approaches to treat corneal injuries have been relatively limited, and this reality underscores the need for new therapeutics that can combine the utility of eye drop formulation with the regenerative properties of amnion.

Corneal homeostasis involves a set of highly regulated cell behaviors and an organized cascade of mechanisms that work synchronously to maintain a functionally clear corneal tissue that enables vision [7]. As the outermost layer covering the ocular surface, the corneal epithelium is an extremely vulnerable tissue that is susceptible to a range of traumatic insults and diseases originating from chemical, mechanical and environmental caused by accidental or surgical occurrence [8]. Corneal pathologies are extremely painful, and severe ocular trauma may hinder the naturally occurring regenerative abilities of the cornea tissue to restore a healthy epithelial surface [9]. Such a condition may ultimately lead to the occurrence of corneal chronic disease, and loss of vision from a poorly refracting ocular surface [10].

Previous work performed by both this group and others have evaluated the use of silk protein for use in corneal tissue regeneration and ocular surface applications [11–16,17]. Silk protein fibers derived from *Bombyx mori* silkworm cocoon has a multi-millennial history of human use as a medical suture [18]. The fibrous component of silk, termed fibroin protein, is inherently non-toxic and has been studied for use in a wide range of biomedical applications as a scaffold material to support cell growth and tissue regeneration [19]. In addition, solubilized fibroin has been shown to have potentially bio-therapeutic applications in treating variety of diseases, such as diabetes [20], chronic wounds [21], inflammation [22], and physical performance [23].

Recently, this group has developed a soluble derivative of fibroin, termed Silk-Derived Protein (SDP), which is amendable for use in ophthalmic formulations due to the material’s inherent high solubility in aqueous solution and stability profile [18]. Recently, the material’s impact on human corneal limbal-epithelial (hCLE) cultures was assessed and found to have enhancing effects on cell migration, adhesion and proliferation [24]. Here, for the first time, a previously described rabbit corneal injury model was utilized to evaluate the impact of aqueous SDP in an eye drop format has on the ocular wound healing response [25].

## Results

### Formulation of SDP eye drops and fluorescein staining of rabbit corneal wounds

**Fig 1:**
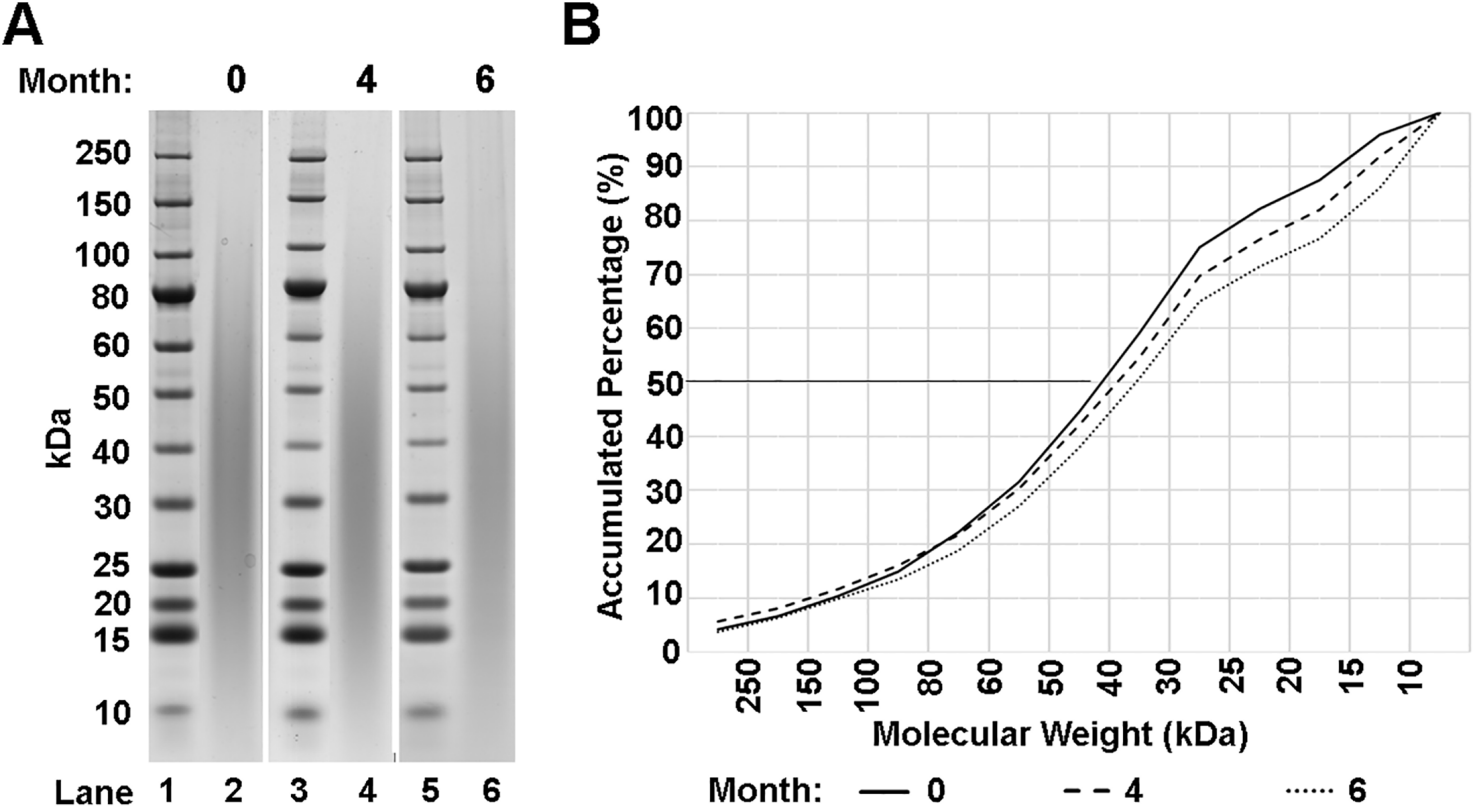
MWD and protein stability analysis. (A) Representative SDS-PAGE gels of aqueous SDP after 0, 4, or 6 months (lanes 2, 4, and 6 respectively) demonstrate overlapping protein compositions (grey region) of like molecular weights. Lanes 1, 3, and 5 depict molecular markers of indicated weights (sized on left in kilodaltons, kDa). (B) Summary graphs derived from electrophoresis histograms of SDP solutions at 0, 4, or 6 months. Protein MWD from 10–250 kDa were evaluated by densitometry and plotted as accumulated percentages of the total protein (0–100%). Average molecular weight is calculated at the 50% accumulation mark, at which point molecular weight is derived from the corresponding intersection on the X-axis.

To determine the longitudinal stability of aqueous SDP, a hermetically sealed SDP solution was stored at 4°C for up to 6 months and then evaluated by visual inspection and for molecular weight distribution by sodium dodecyl sulfate polyacrylamide gel electrophoresis (SDS-PAGE). Visual inspection of the SDP solution revealed all stability sample time points remained free of visual aggregates and microbial contamination during the course of the 6-month stability study (data not shown). Solution samples were drawn from each time point, and SDS-PAGE analysis was performed to determine the molecular weight distribution (MWD) and material stability over time (Fig 1A). Quantitative analysis of the SDS-PAGE results revealed a marginal downward shift in the MWD over the 6-month testing period (Fig 1B). The average molecular weight of the SDP solutions, as determined by gel densitometry, was 42, 38 and 36 kDa at 0, 4 and 6 months, respectively. Distinct protein banding of the SDP was absent as the protein distribution was spread over a broad MWD range of 5 to 250 kDa for each time point, as similarly observed in previous studies using enhanced processing conditions on fibroin protein solutions [26].

**Fig 2:**
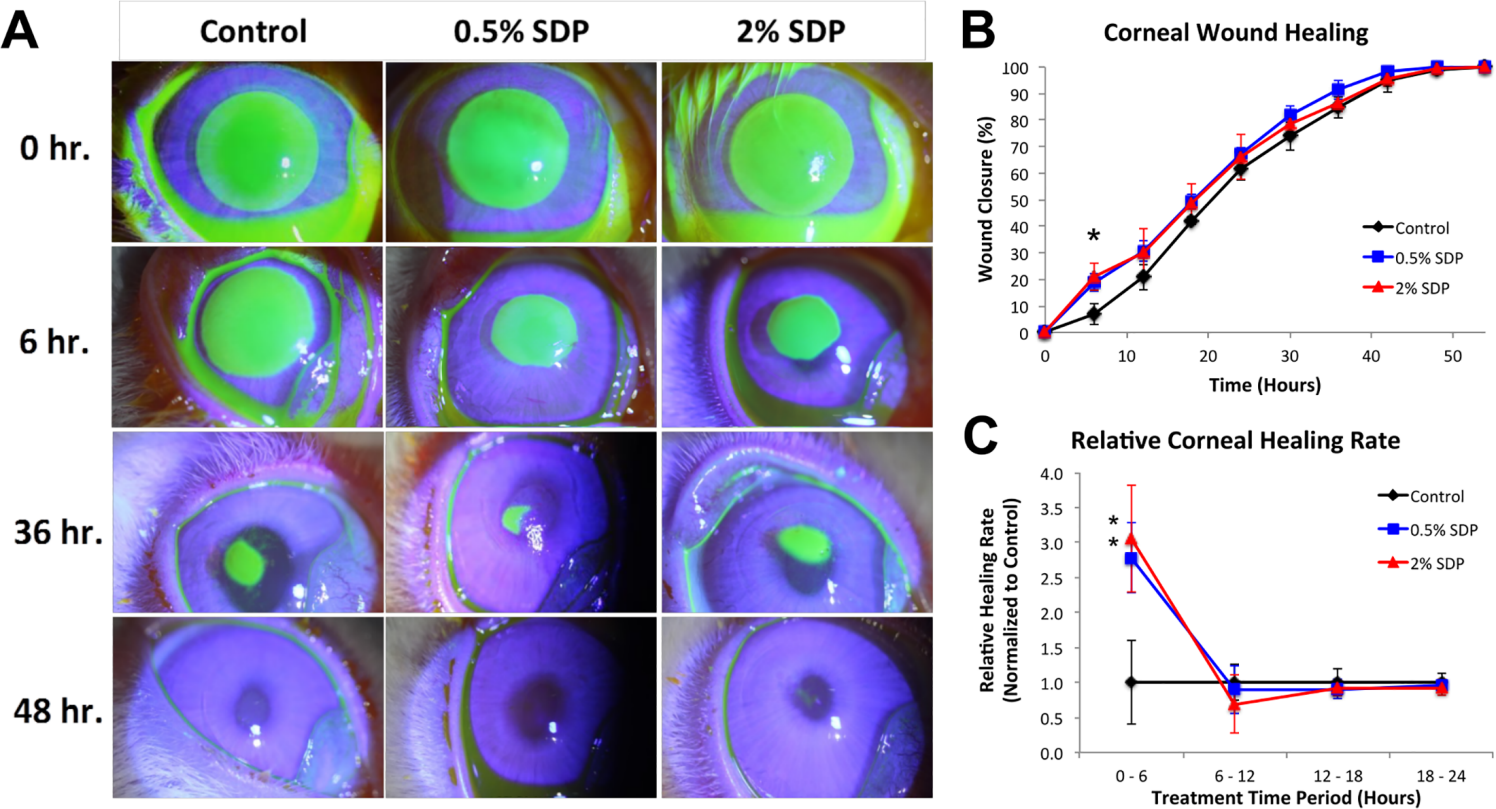
Evaluation of rabbit corneal epithelial wound healing in the presence or absence of SDP. (A) Representative post-surgical fluorescein staining taken at indicated time points of rabbit corneas subjected to epithelial debridement and allowed to heal with 4x per day treatment of PBS (Control) or PBS containing SDP at two different concentrations (0.5%, 2%) over a 48-hour period. (B) Summary of wound closure (percent, %) over time on denuded corneas treated with PBS (Control) or PBS containing SDP at two different concentrations (0.5%, 2%). 2.0% SDP induced a significant increase in wound closure at 6 hours vs. control (*p < 0.05 vs. Control, n = 4). (C) Normalized healing rates between 6–24 hours post-debridement demonstrated a significant increase in healing rate with both SDP concentration treatments. (*p < 0.05 vs. Control, n = 4).

Eye drop bottles containing phosphate buffered saline (PBS) or PBS supplemented with SDP were then prepared for respective dosing of surgical control and treatment groups. The SDP demonstrated a high degree of stability when added at 0.5% and 2.0%, as indicated by the lack of protein aggregates and solution clarity upon visual inspection. Rabbit corneas were denuded of their epithelial layer, and no adverse events were reported during surgery. The initial wound areas were found to be highly regular in shape and consistent amongst the various groups (Fig 2A). Rabbits in all treatment groups exhibited >95% corneal surface repair within the first 48-hours post treatment, consistent with previous results observed with a similar animal model [25].

Eye drops were periodically administered to all rabbit corneal wounds over a 72-hour study period post-surgery, and no adverse events were reported during treatment. At the 72-hour harvesting time point, fluorescein staining demonstrated that all rabbits had a fully formed epithelial layer over the injury site. SDP treatment increased epithelial wound closure during the initial phase of healing (0–6 hours) when compared to PBS-treated controls (Fig 2B), enhancing the incipient wound-healing rate up to three-fold (Fig 2C). It should be noted, however, that these accelerated healing rates stabilized to the healing rate of control groups by the conclusion of the experiment.

### SDP treatment enhances tight junction formation in regenerated corneal epithelium

**Fig 3:**
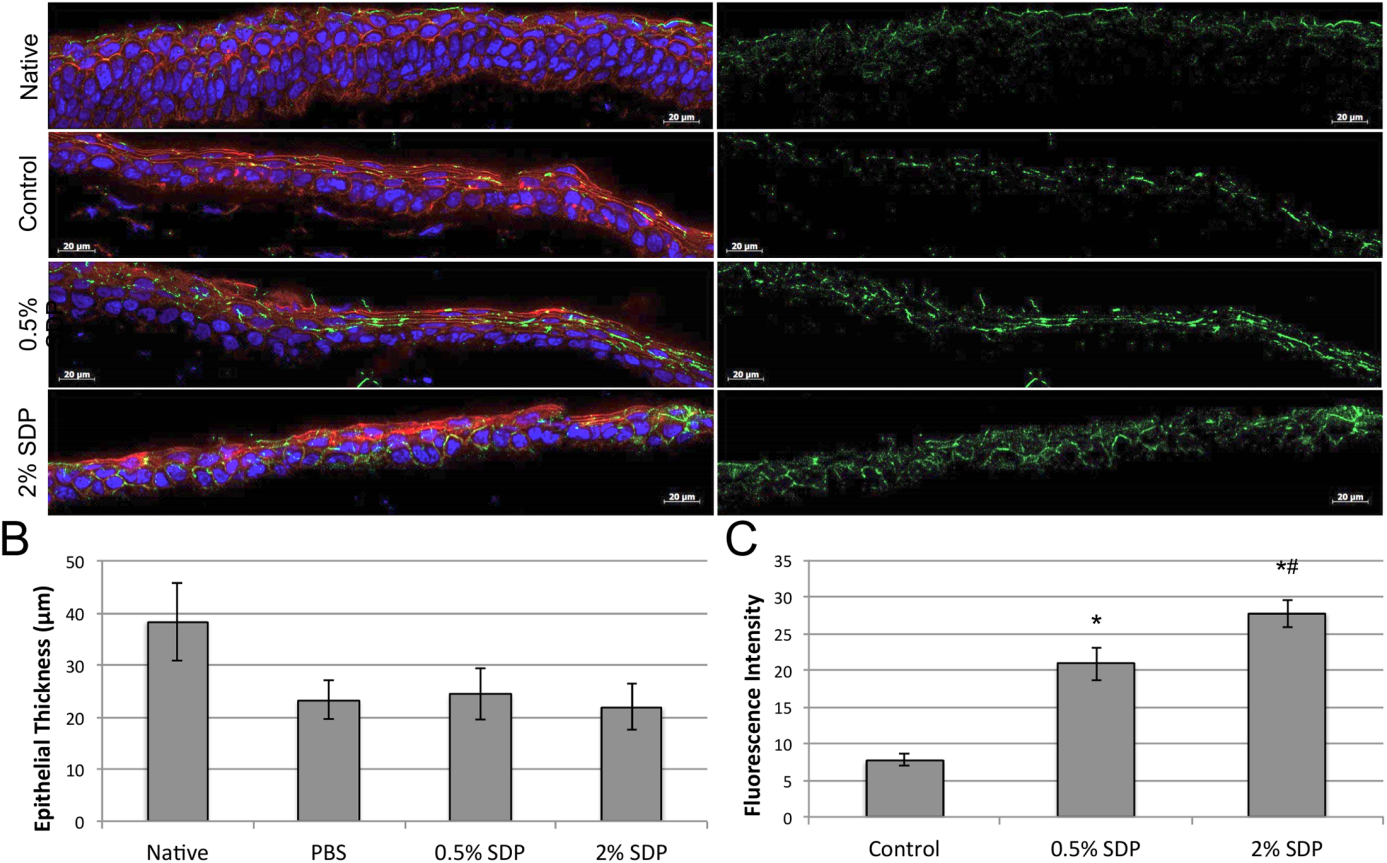
Characterization of rabbit corneal epithelial architecture. (A, left panels) Explanted rabbit cornea cryosections were evaluated by immunofluorescence using DAPI (blue), phalloidin (red), and ZO-1 antibodies (green) for nuclear, actin, and tight junction formation respectively. Staining revealed a multi-cellular epithelial layer had reformed for all treatment groups. (A, right panel) The presence of ZO-1 staining for tight junctions was increased in both SDP-treated groups when compared to PBS-dosed (Control) animals (Scale bar = 20 μm). (B) Summary graph of measured epithelium thickness indicated no significant differences between the various surgical groups, and that native corneal epithelium was less than 50% thicker (n = 4). (C) Summary graph of measured staining fluorescent intensity of ZO-1 in corneas treated with PBS (Control) or SDP concentrations (* p < 0.001 vs. Control; ^#^ p < 0.01 vs. 0.5% SDP, n = 3).

Rabbit corneal wound healing was further evaluated ex-vivo at 72 hours post-surgery through immunofluorescent evaluation of cryosectioned rabbit corneal tissue. Epithelial tissue regeneration was successfully assessed through nuclear (blue), actin cytoskeletal (red), and ZO-1 tight junction (green) staining (Fig 3A, left panels). For all treatment groups a stratified epithelium was reformed and indicated that successful covering of the injury site had occurred. Imaging revealed numerous cell nuclei evenly dispersed through what appeared to be robust actin cytoskeletal architecture suggesting that normal tissue architecture was restored. Reformation of epithelial barrier integrity was further evaluated by tight junction immunostaining of the zona occludin protein (ZO-1) a well characterized tight junctional marker in corneal epithelium [27] ZO-1 staining within the tissue demonstrated that tight junction formation appeared more heavily interspersed within the actin cytoskeletal architecture for SDP treated groups. No significant differences in epithelial layer thickness were observed amongst the surgical groups, and over 50% of the original native epithelial layer thickness was found to recover (Fig 3B).

Tight junction staining was further evaluated independent of actin or nuclear staining (Fig 3A, right panels). Native (untreated) corneas demonstrated positive ZO-1 (green) staining throughout all of the epithelial layers outlining the lateral borders of the squamous epithelial cells comprising the most-superficial layer of the corneal surface, as well as throughout the underlying substratum. Similarly, rabbit corneas that were subjected to surgical debridement, and subsequent PBS treatment showed ZO-1 expression at the cell-cell junctions of the superficial corneal epithelial cells that were newly formed over the wound bed. Rabbit corneas treated with both 5 mg/mL and 20 mg/mL SDP concentrations exhibited positive ZO-1 staining throughout the newly formed superficial epithelial layer, and more closely resembled the native tissue. ZO-1 expression appeared to increase with SDP dosage in treated corneas when compared to PBS controls. Specifically, corneas treated with 0.5% and 2% SDP exhibited a respective 62% and 71% increase in staining intensity relative to the PBS treated corneas (Fig 3C).

### SDP treatment increases epithelial cell proliferation and focal adhesion formation, and reduces presence of MMP-9

**Fig 4:**
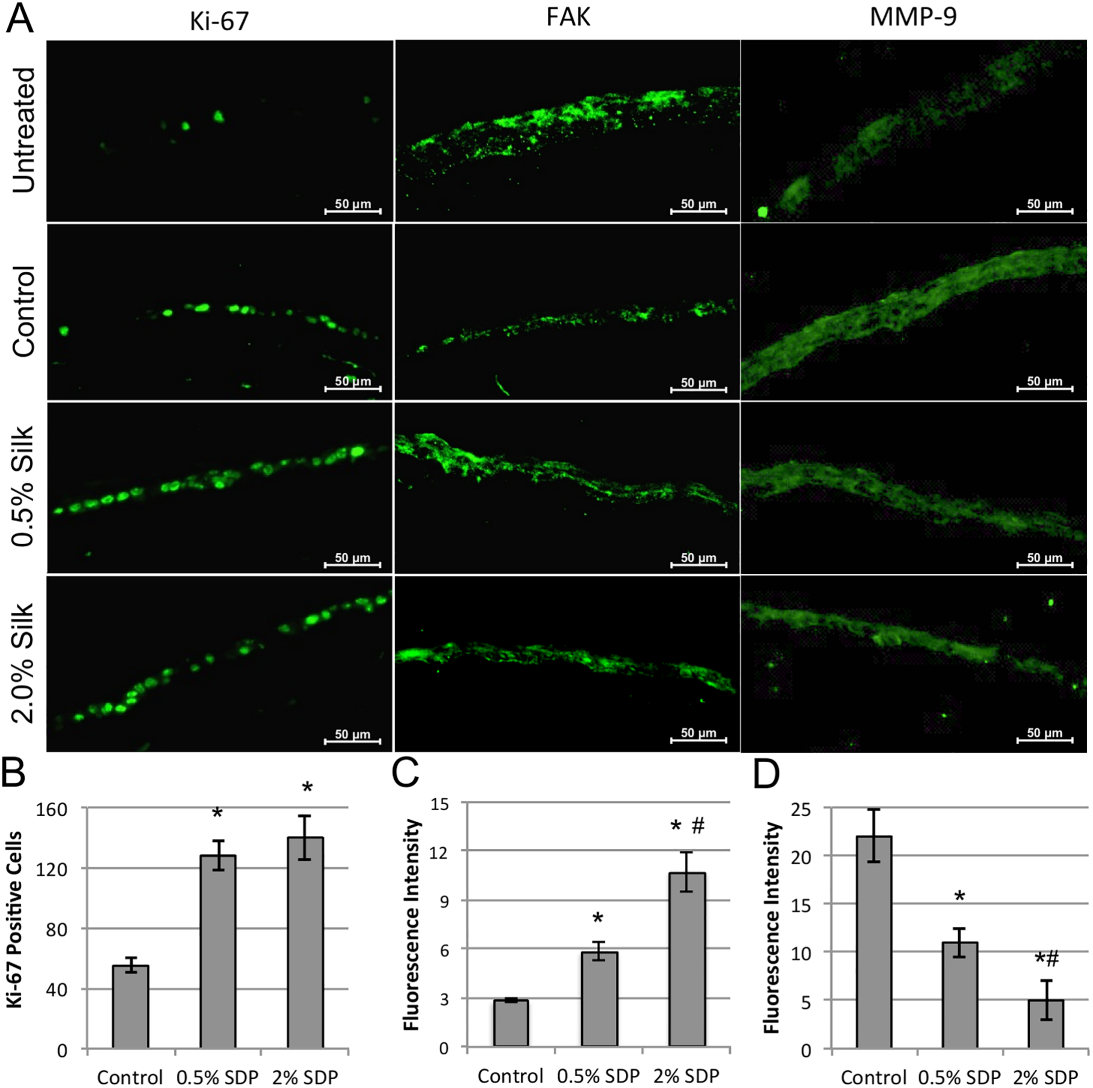
Representative immunohistochemical images of tissue cross-sections obtained from rabbit corneas harvested 72-hours post-surgery. (A, left panels) The presence of staining for the cell proliferation marker Ki-67 increased in denuded PBS treated (Control) animals vs. untreated native corneas, in which Ki-67 staining was further increased with both 0.5% and 2.0% SDP treatment. (A, center panels) FAK staining increased in both SDP treated groups when compared to PBS treated (Control) animals. (A, right panels) MMP-9 staining decreased for both SDP treated groups when compared to PBS treated (Control) animals. (Scale bar = 50 μm). (B) Summary graph of Ki-67 positive cell counts in corneas treated with PBS saline buffer (Control) or SDP concentrations. (C) Summary graph of measured staining intensity of FAK in corneas treated with PBS (Control) or SDP concentrations. (D) Summary graph of measured staining intensity of MMP-9 in corneas treated with PBS (Control) or SDP concentrations. (* p < 0.001 vs. Control; ^#^ p < 0.001 compared to 0.5% SDP, n = 3).

Rabbit corneal wound healing was further evaluated ex-vivo 72 hours post-surgery using immunofluorescence on cryosectioned eyes to investigate cell proliferation, focal adhesion (FA) formation, and matrix enzyme presence. To directly evaluate epithelial proliferation on the corneal surface, antibodies against the nuclear cell cycle marker Ki-67 were used. As anticipated, the untreated native rabbit corneal epithelium exhibited minimal positive Ki-67 staining, and indicates minimal base-line cell proliferation is taking place on the untreated native cornea surface (Fig 4A, left panels). Conversely, epithelial debridement evoked considerable Ki-67 nuclear staining for the PBS treated control group. Critically, treatment with both 0.5% and 2% concentrations of SDP induced a greater increase in cell proliferation as indicated by Ki-67 staining along the entire corneal surface. Quantification of Ki-67 positive cells indicated a significant 2-fold increase in Ki-67 nuclear staining for 2% SDP when compared to PBS treated controls (Fig 4B).

Next, the presence of FA formation was investigated through immunostaining for the classical marker focal adhesion kinase (FAK), to assess epithelial cell attachment to the underlying basement membrane [28]. The untreated native corneal epithelium exhibited strong cell-matrix interactions as demonstrated by robust FAK staining along the entire corneal surface (Fig 4A, center panels). Corneas that underwent epithelial debridement and subsequent treatment with PBS exhibited increased FAK presence throughout the newly formed corneal epithelial layer. Treatment with both 0.5% and 2.0% SDP concentrations induced a dose dependent increase in FAK presence as evidenced by a 51% and 77% increase in staining intensity, respectively, relative to the PBS-treated controls (Fig 4C).

Lastly, immunofluorescent staining was used to localize matrix metalloproteinase 9 (MMP-9), which is a known metalloprotease involved during matrix remodeling and corneal tissue repair [29]. The untreated native corneal epithelium showed minimal MMP-9 staining (Fig 4A, right panels), whereas corneas that were wounded and subsequently treated with PBS exhibited a high level of MMP-9 presence throughout the newly formed corneal epithelial layer. Treatment with both 0.5% and 2.0% SDP concentrations induced a dose-dependent reduction in MMP-9 presence as evidenced by an approximate 50% and 90% reduction in staining intensity, respectively, relative to the PBS-treated controls (Fig 4D).

## Discussion

There is a significant and growing need to develop new alternative therapeutic approaches for treating the injured cornea. Studies to date have indicated that silk-derived materials offer a novel alternative approach for developing new ocular treatments [11,15,16,24] To demonstrate the ability of SDP to enhance wound healing in a multifactorial signaling environment, an established *in vivo* corneal epithelial wound-healing model was utilized in this study. The impact of an SDP eye drop formulation on corneal wound healing was evaluated as compared to a vehicle control on the healing rabbit cornea.

The MWD of 5% SDP protein in DI water was found to remain highly stable due to the lack of aggregate of microbial presence as revealed through visual inspection. In addition, the protein demonstrated minimal degradation over a 6-month period based on MWD measurements. These results for SDP are in contrast to previous regenerated fibroin solution findings, which demonstrate a high degree of formulation instability within 1-month of production [30]. In addition, the lack of banding for SDP is a departure from native fibroin, which typically has strong banding at 390 and 26 kDa for the fibroin heavy and light chains respectively [19]. Instead, SDP shows a broad ‘smear’ pattern that represents the high degree of heterogeneous hydrolyzed protein fragments present within the material. Unlike the production of regenerated fibroin solution, the SDP production process utilizes high pressure and temperature during the dissolution of the protein within LiBr salt solution [18]. This modified process appears to reduce the presence of the fibroin light chain to negligible detection levels by SDS-PAGE analysis, and works to significantly hydrolyze the fibroin heavy chain into smaller molecular weight protein fragments when compared to regenerated fibroin protein. Combined, these effects appear to enhance the material’s stability over time when compared to regenerated fibroin solution.

Rabbit corneas treated with SDP, following surgical epithelial debridement, showed a significant, dose-dependent increase in epithelial migration rate in the acute phase of healing following injury. Accelerated wound closure in the incipient phase of the corneal healing process has potential to minimize the risks associated with wound infection, delay pain onset, and allow for a more robust healing response [7]. However, the overall time to complete wound closure was not significantly different between the SDP treated groups and control. These observations suggest that the soluble SDP may expedite the acute phase of wound healing on the corneal surface, which is then stabilized to baseline control levels overtime. Although the reason behind this apparent biphasic response is unknown, previous work *in vitro* has shown that there is an SDP concentration threshold effect where at a high enough dose cell adhesion begins to overtake gains in cell migration rate [24]. To that end, a similar effect may be happening with SDP treated rabbit corneas at later healing time points that experience multiple doses. More work is required to elucidate the mechanistic origins of this biphasic response observed *in vivo*.

The effective and efficient restoration of epithelial tissue integrity is a clinical hallmark of proper corneal wound healing [31]. Loss of the superficial epithelium from injury leads to the breakdown of tight cell-cell junctions and strong cell-matrix adhesions [32]. Following a corneal injury, an important component to regeneration of a healthy epithelium tissue is the establishment of tight junctions between cells, which are essential for restoring the protective cell-cell barrier to posterior corneal tissue [27]. Organization of the tight junctions between epithelial cells is restored rapidly after injury once the migrating epithelial cell sheets have covered the denuded corneal epithelium. Immunohistochemical evaluation revealed that SDP treatment produced a stratified epithelial layer similar to PBS-treated controls with no indications of aberrant tissue formation. Interestingly, immunostaining results demonstrated that SDP treatment significantly increased positive staining for the tight junction maker ZO-1 throughout the epithelial tissue. This evidence demonstrates that SDP treatment may enhance overall ocular surface barrier integrity through enhanced cell-cell attachment by increasing tight junction formation during the epithelial wound healing process. Further work is required to better understand the extent in which barrier function is increased both for pathogen migration trough the corneal epithelium, and also how potential therapeutic pharmacokinetics across the epithelial surface may be impacted.

Further immunohistochemical analysis of the explanted rabbit cornea tissue revealed the biological impact that SDP had on various components of the wound healing process. During corneal wound healing, the epithelium migrates in a collective sheet sprawling across the injury site, in which cell proliferation is a significant driver of this movement [33]. Treatment with SDP invoked a robust increase in the expression of the proliferative marker Ki-67 in the healed corneal epithelium, which directly demonstrates SDP’s impact on increasing proliferative capacity. Previous work has demonstrated that SDP increases the rate of mitosis for hCLE cells *in vitro* [24]. The observed increase in epithelial proliferation with SDP treatment did not coincide with a significant enhancement in the restoration of epithelium thickness, where all treatment groups demonstrated close to 50% recovery of the original native epithelial layer thickness. This result was anticipated as it is known that for full thickness epithelium restoration post-injury typically takes one month or longer to reach pre-injury measurements [8]. In addition, the overall closure time of the wounds were not different, but instead showed a significant improvement during the incipient phase of the corneal wound healing response. These results may imply that the accelerated rate of epithelial migration exhibited in the early phase of wound healing may be related, in part, to increased epithelial cell proliferation induced by the presence of SDP. Future studies are needed to evaluate the impact of SDP during the later phases of epithelial remodeling to properly evaluate the global effects of SDP on the complete wound healing process.

The maintenance of proper cell-matrix adhesions are critical to the regulation of cell migration and growth during corneal tissue regeneration [34]. Epithelial cells interact with their basement membrane through FA complexes, which act as anchor points that keep the cell attached to the basement membrane [35]. The immunohistochemical analysis above showed a significant increase in punctate FAK staining with SDP treatment. These findings suggest that the presence of SDP facilitates an increase in the cell adhesive interactions with the surrounding and underlying corneal matrix. With the robust increase in FA formation, the regenerated epithelium could be more tightly anchored to the basement membrane and retard migration. This may, in part, explain why at later stages of the rabbit cornea re-epithelialization process there was a noted decrease in wound healing rate relative to untreated controls as the epithelium is more anchored to the respective tissue surface. Similar results in reduced cell migration were shown previously for hCLE cultures [24]. In turn, this may indicate an increased strength of epithelial attachments, which are known to reduce the onset of deleterious persistent epithelial defects (PEDs) and recurring corneal erosions, which are both painful and vision threatening pathologies [32]. Further work is required to elucidate if enhanced FA formations would drive improved clinical outcomes in suffering patients.

Corneal epithelial remodeling also requires the enzymatic degradation of damaged epithelium tissue that is no longer functional [32]. One of the most prevalent proteases in the tear film is MMP-9, in which over-expression of this enzyme has been implicated in a number of pro-inflammatory ocular disease states, and is a known driver of dry eye disease symptoms [36]. Furthermore, MMP-9 is known to actively degrade both tight junctions and focal adhesion formations within a tissue matrix [29,37] It was found that SDP treated rabbit corneas had a significant and dose dependent reduction in resident MMP-9 expression when compared to both untreated native and PBS treated control animals. Notably, it was observed that 2% SDP reduced the presence of MMP-9 staining by nearly an order of magnitude when compared to PBS-treated corneas. The reduction in resident MMP-9 may play a role in the observed increase of both tight junctions and FAs present in SDP treated rabbit corneas as there would be fewer proteases present to degrade these structures. In addition, previous work has shown that soluble fibroin protein reduces MMP-9 gene expression and also act as a substrate for MMP targeted degradation [38,39]. The combined data indicate that SDP treatment appears to reduce MMP-9 presence in the corneal tissue; however, more work is required to determine the cell signaling mechanisms impacted by SDP to reduce tissue presence.

The results from this study demonstrate that the presence of SDP during the corneal epithelial wound healing process provides potentially therapeutic benefits relevant to ophthalmic clinical applications. Future research efforts will be devoted to identifying cell-signaling pathways impacted by SDP, and further characterize how SDP biochemical composition relates to therapeutic mechanisms involved with corneal wound healing responses. Collectively, these initial findings offer compelling evidence that SDP may offer a novel approach for the development of a new ophthalmic therapy for the treatment of ocular surfaces injuries and pathologies. In particular, SDP may find utility in the treatment of recurring corneal erosions or PEDs where current therapies are lacking. In addition, the potential for SDP to reduce resident MMP-9 may also suggest a role for applications in the treatment of dry eye related symptoms. Future efforts will continue to develop SDP for use in the ophthalmic clinical setting with the hope of demonstrating positive outcomes for suffering patients worldwide.

## Materials and methods

### SDP production

*Bombyx mori* silkworm cocoons were purchased from Tajima Shoji Co. (Yokohama, Japan). Silk solution was prepared by cutting 5 g of cocoons into thirds. The cocoons were then boiled in 2 L of 0.03M Na_2_CO_3_ (Sigma-Aldrich) for 45 minutes to remove the sericin protein. After four rinses in deionized water the extracted silk fibroin fibers were dried at room temperature overnight. The dried silk fibroin fibers were then dissolved in a concentrated solution of 54% wt./vol. LiBr solution (Sigma-Aldrich) for 2 hours at 60°C. Then, the solution was autoclaved at 121°C under 15 PSI for 30 minutes. The autoclaved SDP solution was then dialyzed against a 200x volume of water using Snake-Skin dialysis tubing (Thermo Fisher Scientific, Inc.) with a 3,500 molecular weight cut-off (MWCO) for 48-hours and six water exchanges at 1, 4, 8, 12, 12, and 12 hour intervals. The dialyzed solution was then centrifuged twice at 10,000 G for 20 minutes to remove impurities by decanting the supernatant each time. Protein concentration was then calculated by measuring the weight loss on drying of 1 mL samples of SDP solution (n = 3). The solution was then diluted to a 5 wt./vol. % (50 mg/mL) concentration using sterile water and stored at 4°C until use. Sterile 1x PBS solutions at pH 7.4 were prepared using deionized water at both a 10x and 5x concentration with commercially available powder tablets (Sigma-Aldrich), which were then added to the SDP solution to produce 0.5 wt./vol. % (5 mg/mL) and 2 wt./vol. % (20 mg/mL) concentrations of protein, respectively. The solution was then aseptically transferred to a sterile 10 cc volume eye drop bottle (US Plastic Corporation, Lima, OH) for experimental use.

### SDP MWD characterization and stability analysis

The MWD of SDP in DI water was evaluated through visual inspection and SDS-PAGE analysis. Samples of 5% SDP solution were aseptically prepared for 0, 4, and 6-month stability study pull-points by first sterile filtering the solution through a 0.2 polyethersulfone (PES) syringe filter (Sartorius Stedim, Inc.) into 50 mL polypropylene conical vials (VWR, Inc.) within a class 100, clean bench, work area. At each time point samples were visually inspected for aggregate formations or microbial contamination. For SDS-PAGE analysis, 15 μg of total protein content was mixed with sample buffer containing sodium dodecyl sulfate and dithiothreitol (Bio-Rad, Inc.) to remove any secondary folding structures and disulfide bonds, respectively, and then heated to 70°C for 5 minutes. Mixtures were loaded adjacent to a 10–250 kDa molecular weight ladder (New England BioLabs, Ipswich, MA) onto pre-cast, 4–12% polyacrylamide gels containing Bis-Tris buffer salts (Thermo Fisher Scientific, Inc.), and then exposed to 120V electric field for 90 minutes on a Bio-Rad PowerPac Power supply (Bio-Rad, Inc.). Gels were treated with 50% methanol, 10% acetic acid, and 40% DI water for 10 minutes. Gels were then removed and placed in a 20% methanol, 55% DI water, and 25% colloidal blue stain (Colloidal Blue Staining Kit, Invitrogen, Inc.) stain for 12 hours to stain proteins, followed by 6 hours of destaining in deionized water. Rinsed gels were scanned on a Bio-Rad GS-800 Calibrated Densitometer (Bio-Rad, Inc.). Images were then imported into ImageJ (NIH, USA) and analyzed. The proportion of the protein between each ladder band was cumulatively totaled and the number average (50% mark of cumulative total) was calculated as the Average Molecular Weigh (AMW).

### Rabbit corneal injury model

All animals were handled according to the ARVO Statement for the Use of Animals in Ophthalmic and Visual Research, under protocols approved by the Institutional Animal Care and Use Committee at the Weill Cornell Medical College. Twelve, 8–10 week old New Zealand white rabbits were used to evaluate the capability of SDP to enhance wound healing *in vivo*. Rabbits were anesthetized with intramuscular injections of 35–50 mg/kg ketamine, 5–7.5 mg/kg xylazine, and 0.75 mg/kg acepromazine. Topical proparacaine 0.5 wt./vol. % eye drops were also used as supplemental anesthesia. A #15 Bard-Parker blade was then used to remove a 7 mm diameter section of the central corneal epithelium, from only one eye, to create a void in the epithelial surface down to the epithelial basement membrane and stromal tissue interface. Subsequently, the rabbits were divided into three treatment groups, where the wounded corneal surface was treated with either 200 μl of sterile phosphate buffered saline (PBS, pH 7.4, vehicle treatment), 0.5 wt./vol. % (0.5%) or 2.0 wt./vol. % (2%) SDP solution in PBS. The treatments were administered topically to the wounded eyes, along with topical moxifloxacin antibiotic drops (Vigamox, Alcon, Inc.), immediately following surgery and subsequent administrations at 6-hour intervals until complete epithelial closure had occurred. Throughout the healing process, rabbits were closely monitored for evidence of distress or infection, and epithelial wound closure was examined every 6 hours by applying 50 μL of topical fluorescein solution (Sigma-Aldrich) to the injured cornea and imaging the wound using slit lamp photography under cobalt blue illumination. Fluorescein staining is a common technique and a widely used method to assess corneal epithelial defects in rabbit corneal injury models, and there have been no previously reported harmful effects on the corneal epithelium when the fluorescein is administered at frequent intervals [40].

### Wound healing data retrieval and analysis

Following image acquisition, images were analyzed using ImageJ software (Ver. 1.48, NIH). Wounds were traced and measured at each time point using ImageJ software (NIH, Bethesda, MD), and wound closure as a function of time was assessed for each treatment group in triplicate. Percent healing (H_%_) was calculated as the wound area as follows:

$$H_{\%}=\frac{\left(A_{0}-A_{x}\right)}{A_{0}}\times 100$$

Where, A_*x*_ indicates the wound area at a given time point and A_0_ indicates the initial wound area. Both the analysis and calculations for percent wound healing were performed by a masked observer who was not present during the initial surgery or during the time of treatment. Relative wound healing rate (R_H_) was calculated as follows:

$$R_{H}=\frac{\left(A_{1}-A_{2})/(t_{2}-t_{1}\right)}{\left(A_{C1}-A_{C2})/(t_{C2}-t_{C1}\right)}$$

Where, A_1_ indicates the wound area at a given time point and A_2_ indicates the wound area at the time point immediately succeeding the previous measurement, while t_1_ and t_2_ indicate the respective time points for each measurement. And A_C2_ indicates the control wound area at a given time point and A_C2_ indicates the control wound area at the time point immediately succeeding the previous measurement, while t_C1_ and t_C2_ indicate the respective time points for each measurement.

### Rabbit cornea tissue retrieval and immunohistochemistry

Animals from each treatment group were sacrificed immediately after wound healing was completed after 72 hours post surgery, using an overdose of pentobarbital (150 mg/kg) administered into the ear vein, and the corneas from each treatment group were enucleated, excised and fixed immediately in 2 wt./vol. % paraformaldehyde for 40 minutes (Electron Microscopy Sciences, Hatfield, PA, USA). Corneas from the contralateral eyes, which did not undergo surgical debridement, were also harvested, and fixed, to serve as negative controls for the wound healing process. The fixed corneas were subsequently washed three times in PBS for 5 minutes each, and then placed in 30 wt./vol. % sucrose overnight at 4°C before embedding in Tissue-TEK O.C.T (Sakura Finetek USA Inc., Torrance, CA, USA) and frozen at -80°C for cryo-sectioning. Ten-micron thick cross-sections, through the center of the cornea, were obtained and mounted on Superfrost-plus glass slides (Thermo Fisher Scientific, Inc.) for immunohistochemical staining and analysis. Samples were washed three times in PBS and then incubated in blocking buffer containing 1 wt./vol. % BSA (Sigma-Aldrich), 0.25 wt./vol. % Triton-X-100 (Sigma-Aldrich), and 2.5 wt./vol. % goat serum in 1X PBS, for 1 hour at room temperature. After blocking, samples were incubated with murine primary antibody solutions (1:100) for either Ki-67 (M724001-2, Dako, Denmark), FAK (FM1211, ECM-Biosciences, Versailles, KY, USA), ZO-1 (R.40.76, Chemicon, USA), or MMP-9 (ab58803, Abcam PLC, Cambridge, UK) overnight at 4°C. Subsequently, the samples were rinsed thoroughly with PBS and then incubated with Alexa Fluor 488 Green goat anti-mouse secondary antibody (ab150113, Abcam PLC, Cambridge, UK) at a 1:500 dilution for 1 hour at room temperature, protected from light. Samples were also stained with Alexa Fluor^®^ 568 phalloidin (Thermo Fisher Scientific, Inc.) at a 1:20 dilution for 20 minutes at room temperature, protected from light. After washing with PBS, Samples were mounted with VECTASHIELD^™^ Mounting Medium with DAPI (Vector Laboratories, Burlingame, CA, USA) and covered with a glass coverslip.

### Fluorescent image analysis

Fluorescent images were taken using a 63x objective utilizing a 1.6 Optivar optic. Z-stack images (10–25 layer range) were captured at 0.25 μm slices using DAPI, green fluorescent protein and Texas Red filter channels. Image deconvolution was performed on each z-stack using 3D Huygens Deconvolution Software (Scientific Volume Imaging BV, The Netherlands) to assist with attenuating background fluorescence. A total of 40 iterations were performed employing the software’s classic maximum likelihood estimation algorithm for each z-stack, as it was found that increasing the number of iterations had a minimal effect on improving image quality. All other settings were left at the manufacturer’s default settings. Images were produced using maximum intensity projection (MIP) algorithm included in the software, where MIP threshold levels were first determined by default manufacturer’s settings for control corneal tissue to establish a relative fluorescent intensity threshold for each channel. Then, native and SDP treated cornea groups were imaged using these same threshold settings to allow for group comparisons (n = 3) of fluorescent image intensities. Next, fluorescence intensity of each image was measured using ImageJ software (NIH, Ver. 1.48, NIH) by subtracting the mean integrated color densities of a non-fluorescing region from the traced fluorescent region to eliminate background. Fluorescent intensity values amongst the different groups were then calculated as described in the Statistical Analysis section below, and like-stained sample values were plotted together to compare relative fluorescent intensities between groups using Excel Software (Ver. 14.6.7, Microsoft, Inc.).

### Statistical analysis

For all experiments result averages and standard deviations were calculated. In addition, for all experiments, two-way ANOVA analysis was performed amongst groups, and ad hoc Student t-tests were performed to determine p-values and statistical significance using Excel Software (Ver. 14.6.7, Microsoft, Inc.).

## Supporting information

S1 File Excel spreadsheet data files for manuscript figures. Files include raw data for Fig 1 SDS-PAGE analysis of SDP MWD, Fig 2 measurements and calculations for rabbit wound healing, Fig 3 data for corneal epithelial thickness and ZO-1 fluorescence staining intensity measurements, and Fig 4 data for Ki-67, FAK, and MMP-9 fluorescence intensity staining intensity measurements.
(ZIP)
